# General and abdominal obesity in adults living in a rural area in Southern Brazil

**DOI:** 10.11606/S1518-8787.2018052000264

**Published:** 2018-09-13

**Authors:** Thais Martins-Silva, Christian Loret de Mola, Juliana dos Santos Vaz, Luciana Tovo-Rodrigues

**Affiliations:** I Universidade Federal de Pelotas . Programa de Pós-Graduação em Epidemiologia . Pelotas , RS , Brasil; II Universidade Federal de Pelotas . Faculdade de Enfermagem . Pelotas , RS , Brasil; III Universidade Federal de Pelotas . Faculdade de Nutrição . Pelotas , RS , Brasil

**Keywords:** Adult, Obesity, Obesity, Abdominal, Epidemiology, Body Mass Index, Waist Circumference, Risk Factors, Rural Population, Adulto, Obesidade, Obesidade Abdominal, Epidemiologia, Índice de Massa Corporal, Circunferência da Cintura, Fatores de Risco, População Rural

## Abstract

**OBJECTIVE:**

To evaluate the prevalence of general and abdominal obesity and the concomitant presence of both outcomes and their determinants among adults living in a rural area.

**METHODS:**

This cross-sectional, population-based study was carried out in a medium-sized city in the southern region of Brazil. We evaluated three outcomes: general obesity (body mass index ≥ 30 kg/m ^2^ ), abdominal obesity (waist circumference ≥ 102 cm and ≥ 88 cm in men and women, respectively), and concomitant obesities, classified as: no risk, only one risk factor, and aggregate factors. We performed crude and adjusted Poisson regression analyses for each obesity outcome and multinomial logistic regression for metabolic risk. We considered demographic and socioeconomic characteristics as independent variables.

**RESULTS:**

A total of 1,433 individuals were included in the study. Of them, 29.5% presented general obesity and 37.8% presented abdominal obesity. We observed the presence of a risk factor in 15.8% of the sample, while 25.8% presented aggregate factors. The risk of general and abdominal obesity and concomitant outcomes increased significantly with age in both sexes. Richer men were at increased risk for general obesity (PR = 1.7; 95%CI 1.0–2.9), abdominal obesity (PR = 1.8; 95%CI 1.1–2.9), and aggregate factors (OR = 1.9; 95%CI 1.4–5.8). An education level of twelve years or more was a protective factor for women in relation to abdominal obesity (PR = 0.4; 95%CI 0.2–0.8) and aggregate factors (OR = 0.2; 95%CI 0.05–0.7). Rural activity reduced the risk of general obesity (PR = 0.6; 95%CI 0.5–0.8) and aggregate factors (OR = 0.5; 95%CI 0.3–0.8) in women, and the risk of abdominal obesity (PR = 0.6; 95%CI 0.5–0.8) and presence of a risk factor (OR = 0.5; 95%CI 0.3–0.7) in men. Skin color and time lived in rural areas were not statistically associated with the outcomes studied.

**CONCLUSIONS:**

We observed high prevalences of general and abdominal obesity in this population, which is consistent with the values found in urban populations. However, rural activities were a protective factor for obesity outcomes.

## RESUMO

**OBJETIVO:**

Avaliar a prevalência de obesidade geral, abdominal e concomitância de ambos os desfechos e seus determinantes em adultos residentes na zona rural.

**MÉTODOS:**

Este estudo transversal de base populacional foi conduzido em município de médio porte da região Sul do Brasil. Três desfechos foram avaliados: obesidade geral (índice de massa corporal ≥ 30 kg/m ^2^ ), abdominal (circunferência da cintura ≥ 102 cm e ≥ 88 cm em homens e mulheres, respectivamente) e concomitância de ambas, classificada em: sem risco; apenas um fator de risco; e fatores agregados. Foram realizadas análises brutas e ajustadas por regressão de *Poisson* para cada desfecho de obesidade e regressão logística multinomial para o risco metabólico. Características demográficas e socioeconômicas foram consideradas como variáveis independentes.

**RESULTADOS:**

Foram incluídos no estudo 1.433 indivíduos. Desses, 29,5% apresentaram obesidade geral e 37,8%, abdominal. A presença de um fator de risco foi observada em 15,8% da amostra, enquanto 25,8% apresentaram fatores agregados. O risco de obesidade geral, abdominal e a concomitância dos desfechos aumentaram significativamente com a idade, em ambos os sexos. Homens mais ricos apresentaram risco aumentado para obesidade geral (RP = 1,7; IC95% 1,0–2,9), abdominal (RP = 1,8; IC95% 1,1–2,9) e fatores agregados (RO = 1,9; IC95% 1,4–5,8). Ter 12 anos ou mais de estudo se mostrou fator de proteção para mulheres em relação à obesidade abdominal (RP=0,4; IC95% 0,2–0,8) e fatores agregados (RO = 0,2; IC95% 0,05–0,7). Realizar atividade rural reduziu o risco de obesidade geral (RP = 0,6; IC95% 0,5–0,8) e fatores agregados (RO = 0,5; IC95% 0,3–0,8) em mulheres, e de obesidade abdominal (RP = 0,6; IC95% 0,5–0,8) e presença de um fator de risco (RO = 0,5; IC95% 0,3–0,7), em homens. Cor da pele e tempo de vida residido na zona rural não foram estatisticamente associados aos desfechos estudados.

**CONCLUSÕES:**

Altas prevalências de obesidade geral e abdominal foram observadas nessa população, condizentes com os valores encontrados em populações urbanas. Entretanto, a realização de atividades rurais mostrou-se um fator de proteção para os desfechos de obesidade.

## INTRODUCTION

Overweight and obesity are important modifiable risk factors for chronic non-communicable diseases (NCD) ^1^ and were attributed to 3.4 million deaths worldwide in 2010 ^2^ .

Considered as by-products of the processes of accelerated urbanization and globalization, the consumption of high energy density foods, sedentary lifestyle, and obesity showed an increase in their prevalences in the last decades in middle- and low-income countries ^2^ . The lifestyle resulting from urbanization and modernization is also suggested as the cause of the spread of obesity and overweight in rural areas of Brazil ^3^ .

In the South region of Brazil, approximately 15.1% of the total population lives in rural areas ^4^ . These persons have low education level, low income, and difficulty accessing health services ^5^ . Additionally, they have a higher frequency of risk factors, such as smoking, hypertension, and diabetes ^6^ ^,^ ^7^ . However, knowledge about the health conditions and nutritional status of these populations is still limited.

One of the few national surveys to report results from rural areas was the 2008–2009 Household Budget Survey (POF) ^8^ . In this national survey, there was a higher prevalence of general obesity in men living in urban areas (12.3%) compared to those in rural areas (8.8%), while the prevalence observed in women was similar in both areas (approximately 17%) ^8^ . The values observed in the southern region of the country, both in urban and rural areas, were the highest in the country.

Research on the nutritional status of individuals living in rural areas and their determinants is essential, as these data allow the identification of priority groups and behavioral aspects that can be modified. The objective of this study was to identify the prevalence of general and abdominal obesity and the concomitant presence of both outcomes in adults living in a rural area, as well as to evaluate socioeconomic and demographic factors that determine their occurrence.

## METHODS

This study is part of a larger survey entitled “Evaluation of the Health of Adults Living in the Rural Area of the City of Pelotas, State of Rio Grande do Sul, Brazil” (“ *Avaliação da Saúde de Adultos Residentes na Zona Rural do Município de Pelotas, RS* ”), which investigated several health aspects of this population. Pelotas is a medium-sized city located in the southern region of the state of Rio Grande do Sul, Brazil. The rural area of this city has approximately 22,000 inhabitants (6.7% of the total population), divided into eight districts and 50 census tracts, according to the 2010 IBGE Census ^4^ .

It is a cross-sectional, population-based study whose sample consisted of adults (defined by the study as those aged 18 years or over) living in the rural area. A multi-stage sampling was used to visit 720 households and cover the sample size of 1,440 individuals. Twenty-four census tracts were drawn, based on the number of permanent households in each district ^4^ . Within each tract, with satellite images, we identified the household centers, defined as a cluster of at least five houses located within a radius of 1 km from the center. We started selecting the households from the center with the largest number of households and, if we did not reach the number of households needed in this center, we followed to the second largest center and so on until we reached the number of 30 households in each tract. More details can be obtained in the methodological article of this supplement ^9^ .

Trained interviewers collected the data, using tablets and the software REDCap (Research Electronic Data Capture) *.* The questionnaire answered by the participants contained information regarding the household and the individuals living there.

For this study, we used the following anthropometric variables: body mass index (BMI = weight/height ^2^ ) and waist circumference (WC), measured by interviewers previously trained and standardized according to the recommendations proposed by the WHO ^10^ . To measure weight and height, the interviewers used, respectively, an electronic scale with a capacity of 150 kg and an accuracy of 100 g and a mountable adult anthropometer with a maximum value of 2.13 m and a scale of 100 cm. Waist circumference was measured directly on the skin in the narrower region of the body, between the thorax and hip or, if there is no narrower point, at the midpoint between the last rib and the iliac crest, using a inelastic measuring tape with 1.5 m extension.

We considered the outcomes of general obesity, defined by BMI ≥ 30 kg/m ^2^ , and abdominal obesity, adopting the cutoff point of waist circumference greater than or equal to 102 cm for men and 88 cm for women ^10^ . In addition, information on general and abdominal obesity was taken into account for a third outcome, which we used to assess metabolic risk. This variable, mentioned throughout the text as “concomitant outcomes”, was categorized as follows: without risk, only one risk factor (presence of general or abdominal obesity), and aggregate factors (presence of general and abdominal obesity).

To characterize the sample, we used the following demographic and socioeconomic variables: age (in complete years and grouped into the categories 18 to 29, 30 to 44, 45 to 54, 55 to 64, and 65 years or over), skin color (self-reported and categorized as white and non-white), index of goods (quintiles), education level (in full years of study and categorized as 0 to 4, 5 to 8, 9 to 11, and 12 years or more), and marital status (whether or not living with a partner). To measure the effect of the experience and the type of occupational activity carried out in the rural area, we considered two variables: a) percentage of life living in the rural area, obtained by the question “How long have you lived in the rural area of Pelotas?” and categorized as < 50%, 50%–99%, and 100%; and b) occupation related to rural activity, measured by the question “Do you do any rural work, such as those related to farming, livestock, fishing, among others?” and categorized as yes or no.

To evaluate the socioeconomic level, we constructed a variable of index of goods by principal component analysis, which included 22 questions related to the items that were in the house at the time of the interview, among them: piped water, vacuum cleaner, washing machine or dryer and dishwasher, DVD, VCR, refrigerator, microwave, computer (notebook or netbook), television, radio, air conditioner, cable TV or Internet, car or motorcycle. We also included the number of toilets/bathrooms, number of rooms used to sleep, and whether there was a domestic worker. This variable was stratified into quintiles, ranging from the poorest quintile (1st) to the richest quintile (5th).

In order to verify the determinants of general obesity, abdominal obesity, or concomitant outcomes, we constructed a hierarchical conceptual model in which we included the variables of age, skin color, education level, and index of goods in the first level, followed by marital status in the second level, and, lastly, time living in the rural area and occupation related to rural activities in the last level. All variables were kept in the model regardless of their statistical significance. Variables with p value < 0.05 were considered associated with the outcome.

We performed the statistical analyses in the Stata 14.0 program (Stata Corporation, CollegeStation, USA). We considered the cluster sampling effect in all analyses, using the “survey” ( *svy* ) command of the above mentioned program. Furthermore, the analyses were weighted by the number of fixed sampled households reported by the IBGE ^4^ in each evaluated district. We performed crude and adjusted Poisson regression analyses for general and abdominal obesity outcomes, and we obtained prevalence ratio (PR) estimates for each outcome. To evaluate the association between exposure variables and concomitant outcomes, we performed a multinomial logistic regression. For this analysis, we present the odds ratios (OR) for the categories (a) of only one risk factor and (b) for aggregate factors, both compared to the absence of obesity, which was the reference category. The statistical significance of each variable was evaluated by the Wald test for heterogeneity. All the analyses were stratified by sex.

To ensure the quality of the information collected, we trained and selected the interviewers, standardized the anthropometry, restandardized at 60 field days, and carried out a pilot study in a rural area similar to the one sampled. In addition, after the interviews, we applied a reduced version of the questionnaire to 9.7% of the interviewees in order to evaluate the agreement between the responses by the Kappa test. We used the question “Do you know how to read and write?”, resulting in 76.3% repeatability.

This study was approved by the Research Ethics Committee of the Faculdade de Medicina of the Universidade Federal de Pelotas (Process 1.363.979).

## RESULTS

Of the 1,697 individuals identified, 1,519 answered the questionnaire. Of them, 29 individuals were excluded because they were unable to remain standing still, 16 because they were pregnant or had a child younger than six months, and 10 because of a prosthesis or plaster or amputated limbs. This resulted in 1,464 individuals eligible for anthropometry; of them, 31 could not be measured for weight or height and 35 for waist circumference. Thus, the results presented refer to 1,433 individuals with measures of weight and height and 1,429 individuals with measures of waist circumference, corresponding to 84.4% and 84.2% of the initial sample of the survey, respectively. The sample included in the study was mostly female (51.2%), white (85.8%), with up to eight years of education (75.7%), and living with a partner (71.9%). Most (66.9%) lived in the rural area since birth and 34% performed some type of rural activity ( Table 1 ). The mean age of the individuals included in the study was 47 (SD = 0.7) years.

**Table 1:** Distribution of the studied population according to socioeconomic and demographic variables. Rural area of Pelotas, State of Rio Grande do Sul, Brazil, 2016. (n = 1,439)

Variable	Total (n = 1,439)		Men (n = 702)	Women (n = 737)
		
	%		%	%
Age (years)			p = 0.759 ^a^	
18–29	18.5		19.5	17.5
30–44	24.7		24.4	25.0
45–54	21.1		21.1	21.1
55–64	16.4		16.5	16.4
65 or over	19.1		18.4	19.8
Skin color			p = 0.764 ^a^	
White	85.8		85.3	85.8
Non-white	14.3		14.6	14.1
Education level (years)			p < 0.001 ^a^	
0–4	38.3		37.6	38.9
5–8	37.4		40.6	34.5
9–11	19.8		19.2	20.4
12 or more	4.3		12.5	6.0
Quintiles of goods			p = 0.347 ^a^	
1 (poor)	19.6		18.4	20.7
2	19.5		19.7	19.3
3	20.2		20.9	19.6
4	20.2		20.1	20.3
5 (rich)	20.2		20.6	19.8
Living with a partner			p = 0.373 ^a^	
No	28.0		29.0	27.1
Yes	71.9		70.9	72.8
Percentage of life living in the rural area ^b^			p = 0.228 ^a^	
< 50%	19.0		19.1	18.9
50%–99%	14.0		12.5	15.4
100%	66.9		68.3	65.6
Rural activity ^c^			p < 0.001 ^a^	
No	65.1		55.9	73.9
Yes	34.8		44.0	26.0
General obesity ^d^			p < 0.001 ^a^	
No	70.4		74.9	66.1
Yes	29.5		25.0	33.9
Abdominal obesity ^e^			p < 0.001 ^a^	
No	62.1		75.2	49.5
Yes	37.8		24.7	50.4

Concomitant outcomes			p < 0.001 ^a^	
No risk		58.3	68.8	48.3
One risk factor		15.8	12.4	19.0
Aggregate factors		25.8	18.6	32.6

^a^ Pearson’s chi-square test.

^b^ Categorized from the relation between the question: “How long have you lived in the rural area of Pelotas?” and age.

^c^ Categorized from the question: “Do you do any rural work, such as those related to farming, livestock, fishing, among others?”.

^d^ No: body mass index (BMI) < 30 kg/m ^2^ ; Yes: BMI ≥ 30 kg/m ^2^ .

^e^ No: waist circumference (WC) ≤ 102 cm for men and ≤ 88 cm for women; Yes: WC > 102 cm for men and > 88 cm for women.

Mean BMI was 27.7 (SD = 0.3) kg/m ^2^ . Overall, 29.5% had general obesity (25% of men and 33.9% of women) and 37.8% were classified as having abdominal obesity (24.7% of men and 50.4% of women). Regarding the concomitant outcomes, 15.8% (12.4% of men and 19% of women) presented only one risk factor, while 25.8% (18.6% of men and 32.6% of women) presented aggregate factors ( Table 1 ). All outcomes were more prevalent in women (p < 0.001).

Considering general obesity as an outcome ( Table 2 ), risk increased as age increased in both males (p = 0.027) and females (p < 0.001). Compared to the younger age group, those aged 65 years or over presented an approximately twice as high risk for general obesity for men (PR = 2.1, 95%CI 1.33–3.49) and women (PR = 2.5, 95%CI 1.9–3.2). Regarding the index of goods, among men, the richest had a higher risk of being obese than the poorest (PR = 1.7, 95%CI 1.0–2.9), but in women this association was not statistically significant. Performing some rural activity was a protective factor for obesity in women (PR = 0.6, 95%CI 0.5–0.8). Skin color, education level, marital status, and time living in the rural area were not statistically associated with general obesity in both sexes in the adjusted model.

**Table 2:** Crude and adjusted analysis between general obesity and demographic and socioeconomic factors, stratified by sex. Rural area of Pelotas, State of Rio Grande do Sul, Brazil, 2016. (n = 1,433)

Variable	Men		Women	

	_Crude_ PR (95%CI)	_Adjusted_ PR (95%CI)	_Crude_ PR (95%CI)	_Adjusted_ PR (95%CI)
1st level				

Age (years)	p = 0.024 ^a^	p = 0.027 ^a^	p < 0.001 ^a^	p < 0.001 ^a^
18–29	1	1	1	1
30–44	1.46 (0.86–2.45)	1.38 (0.80–2.38)	2.26 (1.63–3.13)	2.25 (1.68–3.00)
45–54	1.72 (1.13–2.60)	1.71 (1.09–2.68)	2.35 (1.75–3.16)	2.35 (1.75–3.16)
55–64	1.78 (1.08–2.94)	1.72 (0.97–3.06)	2.83 (2.10–3.82)	2.74 (2.03–3.72)
65 or over	2.10 (1.31–3.35)	2.16 (1.33–3.49)	2.56 (2.08–3.15)	2.51 (1.94–3.24)
Skin color	p = 0.950 ^a^	p = 0.601 ^a^	p = 0.489 ^a^	p = 0.198 ^a^
White	1	1	1	1
Non-white	1.01 (0.64–1.58)	1.12 (0.70–1.79)	1.09 (0.84–1.41)	1.19 (0.90–1.58)
Years of study	p = 0.237 ^a^	p = 0.233 ^a^	p < 0.001 ^a^	p = 0.168 ^a^
0–4	1	1	1	1
5–8	1.14 (0.89–1.45)	1.23 (0.96–1.58)	0.83 (0.66–1.04)	0.94 (0.74–1.19)
9–11	0.75 (0.47–1.18)	0.91 (0.58–1.42)	0.69 (0.50–0.95)	0.95 (0.66–1.36)
12 or more	1.34 (0.63–2.85)	1.41 (0.59–3.35)	0.35 (0.14–0.85)	0.44 (0.19–1.05)
Quintiles of goods	p = 0.373 ^a^	p = 0.262 ^a^	p = 0.022 ^a^	p = 0.280 ^a^
1 (poor)	1	1	1	1
2	1.65 (0.86–3.17)	1.69 (0.90–3.17)	0.86 (0.56–1.32)	0.91 (0.59–1.41)
3	1.81 (0.99–3.32)	1.83 (1.02–3.28)	1.15 (0.83–1.60)	1.23 (0.87–1.73)
4	1.78 (1.01–3.16)	1.87 (1.09–3.18)	0.89 (0.63–1.25)	1.02 (0.72–1.45)
5 (rich)	1.69 (0.97–2.94)	1.73 (1.01–2.96)	0.75 (0.54–1.03)	0.91 (0.64–1.30)

2nd level				

Living with a partner	p = 0.009 ^a^	p = 0.453 ^a^	p = 0.351 ^a^	p = 0.532 ^a^
No	1	1	1	1
Yes	1.47 (1.10–1.96)	1.12 (0.81–1.57)	1.07 (0.91–1.27)	1.06 (0.87–1.28)

3rd level				

Percentage of life living in the rural area	p = 0.793 ^a^	p = 0.724 ^a^	p = 0.406 ^a^	p = 0.453 ^a^
< 50%	1	1	1	1
50%–99%	1.16 (0.67–2.00)	1.14 (0.68–1.91)	1.24 (0.89–1.73)	1.20 (0.87–1.65)
100%	1.14 (0.76–1.69)	1.16 (0.79–1.71)	1.12 (0.84–1.50)	1.17 (0.84–1.62)
Occupation related to rural activity	p = 0.083 ^a^	p = 0.105 ^a^	p = 0.009 ^a^	p = 0.002 ^a^
No	1	1	1	1
Yes	0.80 (0.63–1.03)	0.79 (0.60–1.05)	0.74 (0.59–0.92)	0.68 (0.54–0.86)

PR: prevalence ratio

^a^ Wald Test.

In relation to abdominal obesity ( Table 3 ), age was also associated in men (p < 0.001) and women (p < 0.001). Older adults were at higher risk than younger individuals (PR = 4.4, 95%CI 2.4–8.1 in men and PR = 3.3, 95%CI 2.3–4.7 in women). Regarding education level, women with 12 years or more of study had a lower risk of abdominal obesity than women with lower education (PR = 0.4, 95%CI 0.2–0.8). Richer men had a higher risk of abdominal obesity than poorer men (PR = 1.8, 95%CI 1.1–2.9). The variables of education and income were not statistically significant in men and women, respectively. Among men, performing some rural activity proved to be a protective factor for abdominal obesity (PR = 0.6, 95%CI 0.5–0.8). Although the same direction was observed in women, the association was not statistically significant. For both sexes, skin color, marital status, and time living in the rural area were not significantly associated with abdominal obesity in the adjusted model.

**Table 3:** Crude and adjusted analysis between abdominal obesity and demographic and socioeconomic factors, stratified by sex. Rural area of Pelotas, State of Rio Grande do Sul, Brazil, 2016. (n = 1,429)

Variable	Men		Women	

	_Crude_ PR (95%CI)	_Adjusted_ PR (95%CI)	_Crude_ PR (95%CI)	_Adjusted_ PR (95%CI)
1st level				

Age (years)	p < 0.001 ^a^	p < 0.001 ^a^	p < 0.001 ^a^	p < 0.001 ^a^
18–29	1	1	1	1
30–44	1.80 (0.86–3.75)	1.68 (0.77–3.64)	2.25 (1.59–3.20)	2.19 (1.57–3.06)
45–54	2.51 (1.42–4.42)	2.39 (1.32–4.32)	2.85 (1.92–4.22)	2.77 (1.91–4.01)
55–64	3.54 (1.90–6.59)	3.30 (1.69–6.47)	3.08 (2.14–4.43)	2.90 (2.08–4.05)
65 or over	4.53 (2.55–8.04)	4.44 (2.44–8.10)	3.65 (2.47–5.38)	3.32 (2.33–4.72)
Skin color	p = 0.546 ^a^	p = 0.988 ^a^	p = 0.507 ^a^	p = 0.085 ^a^
White	1	1	1	1
Non-white	0.82 (0.43–1.57)	1.00 (0.52–1.92)	1.05 (0.89–1.24)	1.15 (0.97–1.36)
Years of study	p = 0.011 ^a^	p = 0.019 ^a^	p < 0.001 ^a^	p = 0.028 ^a^
0–4	1	1	1	1
5–8	1.01 (0.79–1.30)	1.21 (0.96–1.58)	0.70 (0.56–0.88)	0.85 (0.70–1.04)
9–11	0.58 (0.36–0.91)	0.86 (0.58–1.42)	0.56 (0.41–0.75)	0.79 (0.62–1.01)
12 or more	0.77 (0.33–1.79)	0.78 (0.59–3.35)	0.34 (0.17–0.67)	0.42 (0.21–0.85)
Quintiles of goods	p = 0.258 ^a^	p = 0.161 ^a^	p = 0.531 ^a^	p = 0.720 ^a^
1 (poor)	1	1	1	1
2	1.12 (0.67–1.85)	1.19 (0.76–1.88)	0.85 (0.66–1.10)	0.90 (0.70–1.16)
3	1.33 (0.77–2.32)	1.42 (0.91–2.21)	0.94 (0.77–1.14)	1.07 (0.90–1.27)
4	1.53 (0.90–2.58)	1.67 (1.06–2.62)	0.82 (0.64–1.04)	1.00 (0.81–1.24)
5 (rich)	1.61 (0.95–2.74)	1.80 (1.11–2.92)	0.79 (0.58–1.10)	1.05 (0.79–1.41)

2nd level				

Living with a partner	p = 0.013 ^a^	p = 0.525 ^a^	p = 0.771 ^a^	p = 0.346 ^a^
No	1	1	1	1
Yes	1.66 (1.12–2.45)	1.14 (0.74–1.74)	1.02 (0.87–1.18)	1.07 (0.92–1.24)

3rd level				

Percentage of life living in the rural area	p = 0.317 ^a^	p = 0.369 ^a^	p = 0.013 ^a^	p = 0.226 ^a^
< 50%	1	1	1	1
50%–99%	1.39 (0.76–2.54)	1.30 (0.80–2.11)	1.21 (0.96–1.53)	1.07 (0.83–1.38)
100%	1.32 (0.91–1.92)	1.28 (0.88–1.86)	0.94 (0.76–1.15)	0.91 (0.73–1.14)
Rural activity	p = 0.012 ^a^	p = 0.007 ^a^	p = 0.011 ^a^	p = 0.053 ^a^
No	1	1	1	1
Yes	0.69 (0.53–0.91)	0.68 (0.52–0.89)	0.81 (0.69–0.94)	0.82 (0.67–1.00)

PR: prevalence ratio

^a^ Wald Test.

In relation to the concomitant presence of outcomes ( Table 4 ), those aged 65 years or over, compared to younger individuals, presented 5.1 (95%CI 2.5–10.5) times more chance of having both risk among men and 6.9 (95%CI 4.4–10.6) times among women. Regarding education level, the increase in the number of years of study suggests a protective effect for the presence of aggregate factors in men and women, and this is significant for the longest time of study in women (OR = 0.2, 95%CI 0.05–0.7). However, we observed a different pattern for males, considering only one risk factor as outcome. Individuals with education level of 5–8 years had a 70% increase in the chance (95%CI 1.1–2.7) of having only one risk factor compared to those with a lower education level. As for wealth quintiles, richer men presented 1.9 (95%CI 1.4–5.8) times more chance of having the aggregate factors than poorer men. In women, the variable was not associated with any outcome category evaluated. Regarding the performance of rural activities, we observed a protective effect for the outcome categories evaluated in men and women, being it statistically significant for the presence of one risk factor (OR = 0.5, 95%CI 0.3–0.7) in men and for the aggregate factors (OR = 0.5, 95%CI 0.3–0.8) in women. Skin color, marital status, and time living in the rural area were not statistically associated with concomitant outcomes.

**Table 4:** Crude and adjusted analysis between demographic and socioeconomic factors in relation to concomitant outcomes, stratified by sex. Rural area of Pelotas, State of Rio Grande do Sul, Brazil, 2016. (n = 1,430)

Variable	Men				Women			
	
	One risk factor		Aggregate factors		One risk factor		Aggregate factors	
			
	_Crude_ OR (95%CI)	_Adjusted_ OR (95%CI)	_Crude_ OR (95%CI)	_Adjusted_ OR (95%CI)	_Crude_ OR (95%CI)	_Adju_ _sted_ OR (95%CI)	_Crude_ OR (95%CI)	_Adjusted_ OR (95%CI)
1st level								

Age (years)	p = 0.004 ^a^	p = 0.004 ^a^	p = 0.004 ^a^	p = 0.004 ^a^	p < 0.001 ^a^	p < 0.001 ^a^	p < 0.001 ^a^	p < 0.001 ^a^
18–29	1	1	1	1	1	1	1	1
30–44	1.52 (0.74–3.12)	1.59 (0.74–3.42)	1.89 (0.79–4.47)	1.71 (0.67–4.33)	2.55 (1.23–5.28)	2.37 (1.15–4.85)	3.50 (2.23–5.49)	3.38 (2.26–5.06)
45–54	2.54 (1.12–5.75)	3.06 (1.22–7.67)	2.50 (1.23–5.10)	2.36 (1.11–5.01)	5.88 (2.40–14.39)	5.56 (2.19–14.15)	4.57 (2.83–7.36)	4.46 (2.76–7.19)
55–64	2.35 (0.84–6.53)	2.69 (0.86–8.34)	3.65 (1.65–8.07)	3.35 (1.32–8.49)	6.18 (2.86–13.35)	5.52 (2.83–10.76)	6.08 (3.86–9.57)	5.52 (3.43–8.89)
65 or over	3.49 (1.72–7.11)	4.72 (2.11–10.55)	5.12 (2.53–10.38)	5.16 (2.52–10.53)	12.63 (4.98–32.0)	9.91 (4.19–23.42)	7.79 (5.13–11.83)	6.91 (4.47–10.69)
Skin color	p = 0.934 ^a^	p = 0.936 ^a^	p = 0.934 ^a^	p = 0.936 ^a^	p = 0.727 ^a^	p = 0.257 ^a^	p = 0.727 ^a^	p = 0.257 ^a^
White	1	1	1	1	1	1	1	1
Non-white	0.93 (0.46–1.87)	1.12 (0.58–2.17)	0.86 (0.37–2.01)	1.07 (0.42–2.73)	1.12 (0.66–1.90)	1.42 (0.78–2.56)	1.15 (0.77–1.72)	1.44 (0.88–2.34)
Years of study	p = 0.158 ^a^	p = 0.021 ^a^	p = 0.158 ^a^	p = 0.021 ^a^	p < 0.001 ^a^	p = 0.203 ^a^	p < 0.001 ^a^	p = 0.203 ^a^
0–4	1	1	1	1	1	1	1	1
5–8	1.30 (0.82–2.08)	1.78 (1.14–2.79)	1.09 (0.77–1.54)	1.32 (0.87–1.99)	0.40 (0.22–0.71)	0.63 (0.35–1.14)	0.53 (0.32–0.86)	0.75 (0.44–1.26)
9–11	0.81 (0.38–1.75)	1.52 (0.59–3.91)	0.52 (0.27–0.98)	0.75 (0.39–1.43)	0.23 (0.09–0.57)	0.45 (0.18–1.09)	0.37 (0.22–0.64)	0.71 (0.39–1.28)
12 or more	2.31 (0.69–7.72)	3.55 (0.87–14.46)	0.85 (0.23–3.21)	0.83 (0.20–3.49)	0.22 (0.06–0.76)	0.29 (0.07–1.16)	0.13 (0.04–0.44)	0.20 (0.05–0.71)
Quintiles of goods	p = 0.375 ^a^	p = 0.043 ^a^	p = 0.375 ^a^	p = 0.043 ^a^	p = 0.184 ^a^	p = 0.194 ^a^	p = 0.184 ^a^	p = 0.194 ^a^
1	1	1	1	1	1	1	1	1
2	1.02 (0.41–2.50)	1.03 (0.43–2.44)	1.73 (0.74–4.04)	1.90 (0.81–4.46)	0.68 (0.41–1.12)	0.73 (0.38–1.39)	0.72 (0.37–1.40)	0.80 (0.38–1.64)
3	1.30 (0.53–3.18)	1.38 (0.58–3.29)	2.06 (0.92–4.49)	2.25 (1.10–4.61)	0.55 (0.29–1.04)	0.78 (0.42–1.47)	1.05 (0.61–1.80)	1.30 (0.73–2.32)
4	0.98 (0.43–2.24)	0.99 (0.43–2.29)	2.42 (1.08–5.41)	2.84 (1.33–6.09)	0.70 (0.35–1.39)	1.18 (0.61–2.28)	0.68 (0.37–1.23)	0.99 (0.53–1.90)
5	1.17 (0.40–3.46)	1.01 (0.32–3.20)	2.37 (1.13–4.96)	1.90 (1.43–5.85)	0.65 (0.29–1.48)	1.25 (0.51–3.07)	0.58 (0.31–1.08)	0.96 (0.46–1.97)

2nd level								

Living with a partner	p = 0.025 ^a^	p = 0.734 ^a^	p = 0.025 ^a^	p = 0.734 ^a^	p = 0.562 ^a^	p = 0.561 ^a^	p = 0.562 ^a^	p = 0.561 ^a^
No	1	1	1	1	1	1	1	1
Yes	1.62 (0.91–2.86)	1.22 (0.64–2.33)	1.94 (1.20–3.14)	1.21 (0.69–2.11)	0.87 (0.55–1.36)	0.99 (0.58–1.69)	1.11 (0.83–1.48)	1.16 (0.82–1.64)

3rd level								

Percentage of life living in the rural area	p = 0.440 ^a^	p = 0.572 ^a^	p = 0.440 ^a^	p = 0.572 ^a^	p = 0.020 ^a^	p = 0.181 ^a^	p = 0.020 ^a^	p = 0.181 ^a^
< 50%	1	1	1	1	1	1	1	1
50%–99%	1.75 (0.76–4.02)	1.95 (0.82–4.62)	1.33 (0.50–3.51)	1.25 (0.51–3.03)	1.52 (0.74–3.14)	1.08 (0.45–2.54)	1.62 (0.94–2.81)	1.42 (0.73–2.72)
100%	1.01 (0.57–1.77)	1.32 (0.75–2.32)	1.42 (0.82–2.44)	1.34 (0.77–2.33)	0.71 (0.43–1.17)	0.61 (0.34–1.11)	1.04 (0.64–1.69)	1.05 (0.58–1.89)
Rural activity	p = 0.014 ^a^	p = 0.011 ^a^	p = 0.014 ^a^	p = 0.011 ^a^	p = 0.021 ^a^	p = 0.021 ^a^	p = 0.021 ^a^	p = 0.021 ^a^
No	1	1	1	1	1	1	1	1
Yes	0.53 (0.34–0.81)	0.50 (0.31–0.79)	0.70 (0.48–1.02)	0.65 (0.42–1.02)	0.70 (0.50–0.97)	0.78 (0.47–1.29)	0.61 (0.43–0.85)	0.54 (0.35–0.84)

OR: odds ratio

^a^ Wald Test.

## DISCUSSION

The prevalence estimates found in this study were higher than those presented for general obesity in previous studies in rural areas in Brazil: 20.8% in Santa Rosa (State of Rio Grande do Sul), 15% in Cavunge (State of Bahia), and 5.5% to 6.8% in the Vale do Jequitinhonha region (State of Minas Gerais) ^11–15^ . Regarding the prevalence of abdominal obesity, lower values were also reported in rural areas of the state of Minas Gerais (11.6% to 26.7%) ^15^ ^,^ ^16^ .

The differences between the prevalences observed in our study and those reported in the literature can be attributed to factors such as the time elapsed since each study and also the cultural, demographic, and socioeconomic differences observed within the rural area of the study, which may influence the lifestyle of the region. However, high prevalences of obesity for the southern region of the country have already been reported by the 2008–2009 POF ^8^ . Furthermore, the results obtained are consistent with those obtained in the National Health Survey conducted in Brazil in 2013, which indicated high prevalence rates of general obesity in rural areas in men (14.2%) and women (24.3%) in the state of Rio Grande do Sul. Regarding abdominal obesity, prevalences were 24.6% and 54% ^17^ , respectively.

The prevalences observed in this study are similar to those described for the adult population in the urban area of Pelotas. In the adult population, prevalences of 26.1% for general obesity and 30.0% for abdominal obesity were observed in 2010 ^8^ . In older adults, the prevalence of general obesity was 29.9% and abdominal obesity was 50.4% in 2014 ^19^ . When comparing the urban area, we highlight the high prevalence of abdominal obesity in both sexes, especially in women, in the rural area of Pelotas. In this study, half of the women living in the rural area had abdominal obesity, whereas in urban areas, the prevalence reported was 37.5% ^18^ . This suggests that a significant proportion of the women in the rural area of Pelotas are at risk for other NCD, since the accumulation in the abdominal region is related to the increase of adipose tissue in the viscera and it is closely linked to cardiovascular risk factors ^20^ .

The positive association between age, general and abdominal obesity, and the concomitant presence of both observed in this study is consistent with other studies carried out in rural areas ^6^ ^,^ ^21^ and in the urban area of Pelotas ^22^ . It is known that decreased basal metabolic rate, in addition to changes in lifestyle, poor diet, sedentary lifestyle, and redistribution of subcutaneous adipose tissue are characteristics that follow the natural aging process and may represent risk factors for this condition ^14^ ^,^ ^21^ . We also highlight that the high prevalences observed are related to the aging of this population, which could also be a result of the migration of younger individuals to the urban area, which has been already reported in another rural area ^14^ . The pattern of obesity in relation to the socioeconomic level is different between high and low income countries. In high-income countries, a negative association is observed, that is, the higher the socioeconomic level, the lower the prevalence of obesity. On the other hand, in low-income countries the association is positive ^23^ . Although the classification of the Brazilian Association of Research Companies (ABEP) is widely used in urban areas, the specific characteristics of occupation and income in rural areas may compromise classification using this instrument. As a result, we included a variable of ownership of goods of the respondents, analyzed as a proxy for the socioeconomic level. We observed that richer men presented greater risks of general and abdominal obesity. We found higher odds for the presence of aggregate factors in this group. Our findings were consistent with current literature in rural areas ^20^ ^,^ ^24^ ^,^ ^25^ and a Brazilian national survey ^8^ , thus corroborating the initial stages of the epidemiological transition ^24^ .

Regarding education level, the findings suggest that more years of study are a protective factor in women in relation to general and abdominal obesity and aggregate factors. This association was demonstrated in the crude analysis for general obesity considered alone, but the association was lost after adjustment. These findings are in line with the literature, which reports lower prevalences of general and abdominal obesity in women with higher education level. The literature also reports the increase in the prevalence of abdominal obesity with income progression and education level in men ^11^ ^,^ ^20^ . Although this association was not found in this study, the analysis of concomitant outcomes showed a greater chance for the risk of aggregate factors among richer men. This pattern of association has previously been observed in Brazilian urban areas ^18^ .

The rural area presents characteristics related to habits of life different from those of the urban zone, such as higher consumption of family farming products, greater energy expenditure with physical movement in the work, and intense manual work, mainly in the harvest period, which can influence the nutritional status of the population ^21^ ^,^ ^25^ . On the other hand, the modernization and development of the countryside ^26^ , strongly linked to access to service goods that reduce the demand for manual labor in daily tasks, have often been suggested as important contributory factors for the imbalance of the body energy balance and they have been related to the increase in the prevalence of general obesity ^13^ ^,^ ^26^ . The presence of an occupation related to rural activity was a protective factor for general obesity in women and for abdominal obesity in men. Occupation in a rural activity was also a protective factor against the presence of a risk factor in men and against aggregate factors in women, corroborating the plausibility of concentration of greater physical activity in the work domain in men and domestic domain in women, reported previously in Brazilian rural areas ^27^ . Furthermore, the literature shows an association between the degree of rurality and lower BMI values in India and China ^25^ ^,^ ^28^ . However, this study does not demonstrate an effect with statistical significance in the time living in the rural area in the evaluated outcomes. It is possible that differences in the type of colonization, in the eating habits, and in the degree of mechanization in relation to the rural area of Pelotas can explain this difference.

This study presents some limitations. The variable of occupation with a rural activity, although associated with the three outcomes, does not necessarily reflect a causal relationship, given the transversal nature of the study. In addition, the variables of time living in the rural area and rural activity were obtained directly from the participants and not from records of rural production and registered workers. However, the associations between rurality and obesity observed in this study can be used as hypotheses and be further explored in future studies. Regarding anthropometry, although BMI does not distinguish fat mass from lean mass, its use supplemented by the waist circumference measure may offer a more accurate evaluation of the nutritional risk, decreasing the limitations of only using BMI ^29^ .

Despite the limitations, this study has the largest sample from rural areas in Brazil up to date. The high response rate contributes to its internal validity. However, generalizations of the results to other populations should be done carefully, since the rural area studied may not represent the Brazilian rural area as a whole.

In conclusion, our study reveals high prevalences of general obesity, abdominal obesity, and concomitant outcomes in a rural area of Southern Brazil. In general, the associations observed between demographic and socioeconomic factors are similar to the associations in studies carried out in urban areas. However, the performance of rural activities proved to be a protective factor for obesity outcomes. Future work is important to clarify the role of this variable in determining obesity in the rural areas of Brazil.
